# Baseline Gut Microbiota Composition Is Associated With *Schistosoma mansoni* Infection Burden in Rodent Models

**DOI:** 10.3389/fimmu.2020.593838

**Published:** 2020-11-18

**Authors:** Alba Cortés, Simon Clare, Alice Costain, Alexandre Almeida, Catherine McCarthy, Katherine Harcourt, Cordelia Brandt, Charlotte Tolley, James Rooney, Matthew Berriman, Trevor Lawley, Andrew S. MacDonald, Gabriel Rinaldi, Cinzia Cantacessi

**Affiliations:** ^1^ Department of Veterinary Medicine, University of Cambridge, Cambridge, United Kingdom; ^2^ Departament de Farmàcia i Tecnologia Farmacèutica i Parasitologia, Facultat de Farmàcia, Universitat de València, València, Spain; ^3^ Wellcome Sanger Institute, Wellcome Genome Campus, Hinxton, United Kingdom; ^4^ Manchester Collaborative Centre for Inflammation Research, University of Manchester, Manchester, United Kingdom; ^5^ European Bioinformatics Institute (EMBL-EBI), Wellcome Genome Campus, Hinxton, United Kingdom

**Keywords:** helminth-gut microbiota interactions, *Schistosoma mansoni*, human-microbiota associated mouse models, gut microbial diversity, dysbiosis, immune-modulation

## Abstract

In spite of growing evidence supporting the occurrence of complex interactions between *Schistosoma* and gut bacteria in mice and humans, no data is yet available on whether worm-mediated changes in microbiota composition are dependent on the baseline gut microbial profile of the vertebrate host. In addition, the impact of such changes on the susceptibility to, and pathophysiology of, schistosomiasis remains largely unexplored. In this study, mice colonized with gut microbial populations from a human donor (HMA mice), as well as microbiota-wild type (WT) animals, were infected with *Schistosoma mansoni*, and alterations of their gut microbial profiles at 50 days post-infection were compared to those occurring in uninfected HMA and WT rodents, respectively. Significantly higher worm and egg burdens, together with increased specific antibody responses to parasite antigens, were observed in HMA compared to WT mice. These differences were associated to extensive dissimilarities between the gut microbial profiles of each HMA and WT groups of mice at baseline; in particular, the gut microbiota of HMA animals was characterized by low microbial alpha diversity and expanded Proteobacteria, as well as by the absence of putative immunomodulatory bacteria (e.g. *Lactobacillus*). Furthermore, differences in infection-associated changes in gut microbiota composition were observed between HMA and WT mice. Altogether, our findings support the hypothesis that susceptibility to *S.* *mansoni* infection in mice is partially dependent on the composition of the host baseline microbiota. Moreover, this study highlights the applicability of HMA mouse models to address key biological questions on host-parasite-microbiota relationships in human helminthiases.

## Introduction

The study of the interactions between the resident microbiota of the vertebrate gut and both enteric and non-enteric parasitic worms is emerging as a key area of research (1–3). Indeed, over the last decade, several studies performed under both experimental and natural conditions of parasite infections, have provided evidence of quantitative and qualitative changes in populations of gut and/or fecal bacterial communities associated with worm establishment (4, 5). Some of these studies have shown that infection-associated alterations of the host gut microbiota impact on the pathogenesis and pathophysiology of helminthiases, e.g. *via* changes to worm burdens (6) and effects on parasite immune-modulatory properties (6–9). Other investigations suggest that the host gut flora contributes to the gastrointestinal pathology associated with helminth infection (10, 11). Nonetheless, disentangling the immune-molecular mechanisms that underpin these interactions is essential for establishing causal relationships between helminths and the gut microbiota. In addition, such knowledge will assist the development of intervention strategies for parasite control, and/or of novel therapeutics for the prevention and treatment of chronic inflammatory diseases, based on the rational manipulation of the host gut microbiota (12).

The causative agents of the major neglected tropical disease schistosomiasis are blood flukes (i.e. trematodes) of the genus *Schistosoma*, that includes *S. mansoni*, *S. haematobium* and *S. japonicum* among other, less prevalent, species (13). More than 250 million people are estimated to be infected with these parasites worldwide, mainly in tropical impoverished areas (13). In addition to the hepato-intestinal disease associated with *S. mansoni* and *S. japonicum*, and the urogenital pathology caused by *S. haematobium*, which includes bladder cancer, all forms of schistosomiasis are associated with systemic morbidities that include malnutrition, anemia, physical and/or cognitive impairment and stunted development in children (13). Over the last few years, a number of studies have demonstrated that infections by *Schistosoma* spp. are associated with quantitative and qualitative alterations of the gut microbial profiles of both humans (14–18) and experimentally-infected mice (10, 19–21). In particular, experiments conducted in murine models of hepato-intestinal schistosomiasis (i.e. by *S. mansoni* and *S. japonicum*, whose eggs penetrate the intestinal wall and are released into the environment *via* the feces) have suggested that, while both egg-related and -unrelated mechanisms contribute to the interactions between *Schistosoma* parasites and the host gut microbiota, the former exert a greater impact on intestinal microbial communities (19–21). In support of this hypothesis, we have previously reported dramatic changes in the gut microbiota profile of mice experimentally infected with *S. mansoni* cercariae that, while detectable during the pre-patent period, were most evident following the onset of egg-laying (19). In particular, features of microbial dysbiosis, including reduced alpha diversity and expansion of proinflammatory bacteria were observed during the patent phase of infection (19). In addition, Floudas *et al*. (20) have shown that perturbations in gut microbiota composition were more extensive in mice infected with mixed-sex *S. mansoni* than in mice colonized by single-sex (male) worms.

While the causality of *Schistosoma*-microbiome interactions remains unclear, it seems plausible that quantitative and qualitative changes in the host gut flora upon parasite colonization may contribute to the pathophysiology of schistosomiasis (10, 19, 21). However, experimental data on the impact of *Schistosoma* parasites on the host gut microbiota originate from studies conducted in wild type mouse models of infection, and no information is available on possible variations that changes in baseline microbiota communities might introduce into these systems. Determining whether the baseline gut microbiota composition of the vertebrate host affects the magnitude of changes observed upon *Schistosoma* infection is nonetheless pivotal, as it underpins the translatability of findings from mice to humans. In this study, we assessed quantitative and qualitative changes in gut microbiota composition of mice pre-colonized with a microbiome of human origin (i.e. human microbiota-associated [HMA] mice) following experimental infections with *S. mansoni* cercariae, and compared the findings with those observed in wild type (WT) animals. Strikingly, higher worm and egg burdens were observed in HMA mice compared to WT animals, and these findings were associated with specific changes in the microbiota composition of each rodent line.

## Materials and Methods

### Ethics Statement

The life cycle of *S. mansoni* (NMRI strain) is maintained at the Wellcome Sanger Institute (WSI) by breeding and infecting susceptible snails (*Biomphalaria grabrata*) and mice. All mouse infections and regulated procedures described in this study were presented to and approved by the Animal Welfare and Ethical Review Body (AWERB) of the WSI. All experiments were conducted under the Home Office Project Licenses (Procedure Project License - PPL) No. P77E8A062 held by GR, and No. P6D3B94CC held by TL. The AWERB is constituted as required by the UK Animals (Scientific Procedures) Act 1986 Amendment Regulations 2012.

### Generation of Human Microbiota-Associated Mice and Experimental Infections

The HMA mouse line was generated at the WSI. Briefly, fresh feces from a healthy human donor, processed within 1 h from transportation to the laboratory, were homogenized at 100 mg/ml in 1x D-PBS in an anaerobic cabinet (80% CO_2_, 10% H_2_, 10% N_2_). Five male and five female C57BL/6 germ-free mice, bred at the WSI, were inoculated by oral gavage with 200 μl of donor homogenates once a week, for 3 weeks. One week after the final gavage, mice were set up as breeding pairs in a decontaminated positive pressure isolator. Mice used in this study belonged to the 4^th^ generation of breeding animals. All consumables that entered the isolator for maintenance of the colony were autoclaved at 121°C for 15 min. Feces from parental HMA mice (i.e. germ-free transplanted with human feces) were analyzed by 16S rRNA gene amplicon sequencing, in order to confirm engraftment of the human flora (unpublished data). Animals used for experiments were removed from the isolator in sealed ISOcages and maintained on a positive pressure ISOrack (Tecniplast).

A total of 10 HMA and 10 WT (C57BL/6, bred at the WSI) mice were exposed to 80 *S. mansoni* cercariae (*Sm*-exp), as described previously (22), and maintained for 50 days with access to food and water *ad libitum*. Additionally, 6 WT and HMA mice were kept uninfected and used as negative controls (*Sm*-). Experimental infections, with matched uninfected controls, were performed in two independent batches (B1 and B2), as indicated in **Table 1**.

**Table 1:** Numbers of wild type (WT) and human microbiota-associated mice (HMA) successfully infected by *Schistosoma mansoni* and left uninfected, and number of samples from each rodent line processed for downstream parasitological and microbiota analyses.

Mouse line	Experimental infection				N° of mice used for		
	Parasite exposure^a^	N° mice/batch^b^	Outcome of the infection^c^	N° mice/batch	Parasitological analyses	16S rRNA gene sequencing & QIIME2	Calypso
WT	***Sm*-exp**	B1 = 5 B2 = 5	***Sm*+**	B1 = 5	8	8	8
				B2 = 3			
			^*^Sm-	B1 = 0	Excluded from further analysis		
				B2 = 2			
	***Sm*-**	B1 = 3 B2 = 3	***Sm*-**	B1 = 3	6	6 (3xB1 + 3xB2)	5 (2xB1 + 3xB2)
				B2 = 3			
HMA	***Sm*-exp**	B1 = 5 B2 = 5	***Sm*+**	B1 = 5	10	8 (5xB1 + 3xB2)	8
				B2 = 5			
	***Sm*-**	B1 = 3 B2 = 3	***Sm*-**	B1 = 3	6	6 (3xB1 + 3xB2)	6 (3xB1 + 3xB2)
				B2 = 3			

a Mice experimentally exposed to S. mansoni cercariae (Sm-exp) or left uninfected (Sm-).

b Experimental infections were carried out in two independent batches (i.e. B1 and B2); each experiment included uninfected mice (negative controls).

c Mice successfully infected with S. mansoni (Sm+) or left uninfected (Sm-) at 50 days post-cercarial exposure. *Sm- = mice exposed to S. mansoni cercariae from which no adult worms nor parasite eggs were recovered at necropsy.

### Sample Collection and Parasitological Analyses

Serum samples were obtained from blood collected by puncture of the tail vein at days 28, 42, and 49 post-cercarial exposure (p.i.). at days 28, 42, and 49 post-infection. At day 50 p.i., *Sm*-exp mice were euthanized, *S. mansoni* adult worms were recovered by portal perfusion, and livers removed for parasite egg counts (22). Briefly, liver sections from the right lobe were weighed and digested overnight in 3 ml of freshly prepared 4% KOH in 1x PBS, at 37°C under gentle agitation. Eggs were counted in 20 aliquots of 10 µl each, and the number of eggs per gram (EPG) of liver was calculated by extrapolating the mean number of eggs per aliquot to the total volume, and dividing by the weight of liver tissue employed (**Supplementary Table 1**). Female fecundity, i.e. estimation of the number of eggs produced by individual female worms, was assessed by dividing the EPG of liver by the total number of female worms collected from each mouse. Statistically significant differences in worm burdens and EPG of liver between infected (*Sm*+) WT and HMA mice were assessed by unpaired two-tailed t-test, following the application of the Kolmogorov-Smirnov normality test for assessment of normal distribution of worm and egg counts datasets; differences in female fecundity were evaluated by Mann-Whitney test. Control *Sm*- mice were perfused under the same conditions as *Sm*-exp animals and the perfusates were discarded. For microbiota profiling, fecal pellets were collected directly from the colons of *Sm*-exp and *Sm*- mice of each line (i.e. WT and HMA) at necropsy. Samples were transferred to sterile tubes, snap frozen in dry ice, and stored at −80°C until DNA isolation, which was performed within 3 weeks from sample collection.

### Specific Antibody Detection

A subset of mice, including six *Sm*+ and three *Sm*- animals from each HMA and WT groups were randomly selected to investigate the kinetics of *S. mansoni*-specific antibodies. Endotoxin-free soluble egg antigen (SEA) was generated from *S. mansoni* eggs isolated from trypsinized livers of infected mice, as previously described (23). The presence of IgG (total, IgG1, IgG2b, IgG2c and IgG3), and IgM reactive against SEA in mouse serum was determined by ELISA (24). Briefly, 96 well plates were coated with 5 μg/ml SEA overnight, before blocking for 1h at room temperature with 1x PBS containing 1% bovine serum albumin (BSA, Sigma). After blocking, and between each incubation step, plates were washed 5 times with PBS containing 0.05% Tween-20 (Sigma). Fifty microliters of diluted serum (1:1,000; PBS+1% BSA) were added to antigen coated wells and incubated for 1h at room temperature. SEA-specific isotypes were detected using alkaline phosphatase-conjugated goat anti-mouse IgG1, IgG2b, IgG2c, IgG3, or IgM antibodies (Southern Biotech). Bound IgG or IgM were detected by adding liquid p-nitrophenyl phosphate substrate (Southern Biotech), with absorbance read at 405 nm. Additionally, total serum IgE was measured using paired capture and detection antibodies (BD-Biosciences), and quantity was assessed *via* standard curve. Statistically significant differences in serum antibody levels between *Sm*+ and *Sm*- mice of each WT and HMA were determined at each p.i. time point by Mann-Whitney test.

### DNA Isolation and Microbial 16S rRNA Gene Sequencing

Genomic DNA was isolated from fecal samples and (no-DNA template) negative controls, using the PowerSoil DNA Isolation Kit (Qiagen) according to manufacturers’ instructions. A total of 28 samples including eight WT and eight HMA *Sm*+ (selected based on highest DNA concentration and quality), six WT and six HMA *Sm-* mice, as well as four negative controls were processed for high-throughput sequencing of the bacterial 16S rRNA gene (**Table 1**). Sequencing libraries were prepared following the Illumina recommendations for bacterial 16S rRNA library preparation, with minor modifications. Briefly, the V3-V4 region of the bacterial 16S rRNA gene was amplified by conventional PCR using universal primers (25) containing the Illumina adapter overhang nucleotide sequences (Forward: 5’ TCGTCGGCAGCGTCAGATGTGTATAAGAGACAGCCTACGGGNGGCWGCAG; Reverse: 5’ GTCTCGTGGGCTCGGAGATGTGTATAAGAGACAGGACTACHVGGGTATCTAATCC), Q5^®^ NEBNext Hot Start High-Fidelity DNA Polymerase (New England Biolabs), 5 ng/μl of template DNA and the following thermocycling protocol: 98°C for 2 min, 20 cycles of 98°C/15 s - 63°C/30 s - 72°C/30 s, and a final elongation step at 72°C for 5 min. Amplicons were purified using AMPure XP beads (Beckman Coulter) and set up for the index PCR using Q5^®^ NEBNext Hot Start High-Fidelity DNA Polymerase (New England Biolabs) and Nextera XT index primers (Illumina) with thermocycling conditions as follows: 3 min at 95°C, eight cycles of 30 s at 95°C – 30 s at 55°C – 30 s at 72°C, and 5 min at 72°C. The indexed samples were purified using AMPure XP beads, quantified using Qubit™ dsDNA High Sensitivity Kit (Life Technologies) and equal DNA amounts from each sample were pooled. The resulting pooled library was quantified using the NEBNext^®^ Library Quantification Kit for Illumina^®^ (New England Biolabs), and sequenced using the v3 chemistry (2x300 bp paired-end reads, Illumina). DNA isolation and sequencing were performed separately for samples collected from each B1 and B2 mice (**Table 1**). Raw sequence data are available from the European Nucleotide Archive (ENA) database under accession number PRJEB40559, whilst curated data can be accessed *via* MICHELINdb (26).

### Bioinformatics and Statistical Analyses

Paired-end demultiplexed Illumina sequencing reads were imported into the Quantitative Insights Into Microbial Ecology 2 (QIIME 2; 2019.4 distribution, https://qiime2.org) software suite for downstream analysis (27). Sequences were then quality filtered, dereplicated, chimeras identified, and paired-end reads merged in QIIME2 using DADA2 (28); quality filtering was performed using default settings, trimming was set at position 10 (forward and reverse), and truncation lengths were set at 250 and 240 for forward and reverse, respectively. A phylogenetic tree was generated using the align-to-tree-mafft-fasttree pipeline in the q2-phylogeny plugin. Bray-Curtis dissimilarity between samples was calculated using core-metrics-phylogenetic method from the q2-diversity plugin. Classification of Amplicon Sequence Variants (ASVs) was performed using a naïve Bayes algorithm trained using sequences representing the bacterial V3-V4 rRNA region available from the SILVA database (https://www.arb-silva.de/download/archive/qiime; Silva_132-99) (29), and the corresponding taxonomic classifications were obtained using the q2-feature-classifier plugin in QIIME2. The classifier was then employed to assign taxonomic information to representative sequences of each ASV.

Statistical analyses were performed using the Calypso software (cgenome.net/calypso/) (30). Relative abundances of individual microbial taxa were calculated by total sum normalization (TSS). Furthermore, for comparative analyses of taxa abundances between experimental groups, cumulative-sum scaling (CSS) was applied to the ASV table, followed by log2 transformation (CSS+log) to account for the non-normal distribution of taxonomic counts data. Statistical comparisons were performed between samples collected from uninfected WT and HMA mice, as well as between *Sm*+ and *Sm*- samples of each WT and HMA, as follows. Unsupervised Principal Coordinates Analysis (PCoA) on Bray-Curtis dissimilarities and supervised Canonical Correlation Analysis (CCA) were performed using ‘mouse line’ (i.e. WT and HMA) and ‘infection status’ (i.e. *Sm*- and *Sm*+) as explanatory variables, respectively. Beta diversity was calculated using Bray-Curtis dissimilarity and differences between groups were assessed using Analysis of Similarity (ANOSIM) (31). Differences in gut microbiota composition between experimental groups were assessed by ANOVA and by using the Linear discriminant analysis Effect Size (LEfSe) workflow (32). Correlations between differentially abundant bacterial genera and worm burdens or EPG of liver were examined *via* multiple linear regressions by incorporating worm burdens (or EPG of liver) as the dependent variable and bacterial genera as explanatory variables. Fecal microbial alpha diversity (Shannon index), richness and evenness were calculated using the ‘diversity’ and ‘specnumber’ functions of the vegan R package and differences between groups were evaluated by Mann-Whitney test.

Clusters of co-occurring bacteria and their association with *Sm+* or *Sm-* mice in each line (i.e. WT and HMA) were identified by prediction of correlation networks, with microbial genera represented as nodes, genera abundances as node size, and edges representing positive associations among them. Networks were generated by computing associations among microbial taxa using Pearson’s correlation. The resulting pairwise correlations were converted into dissimilarities and used to ordinate the nodes in a two-dimensional plot by PCoA; thus, correlating nodes were located in close proximity, while anti-correlating nodes were placed at distant locations in the network. Taxa abundances were associated with the type of infection using Pearson’s correlation and the nodes were colored based on the strength of this association.

## Results

### Worm and Egg Burdens in HMA *vs.* WT Mice

**Figure 1:**
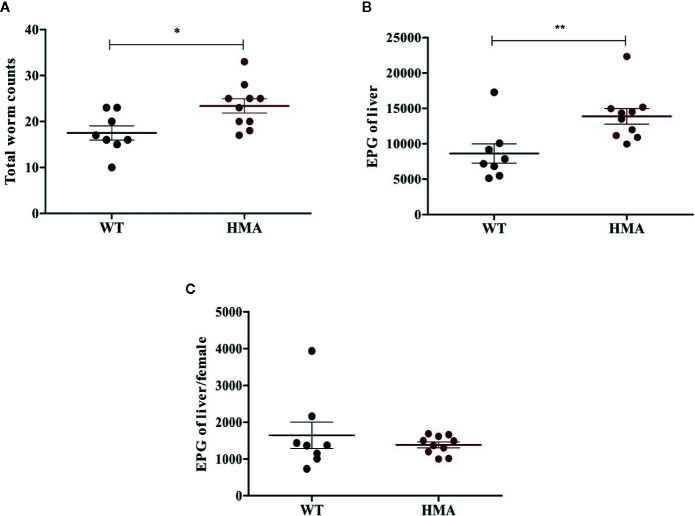
Host microbiome affects susceptibility to *Schistosoma mansoni* infection. Mean number of (± standard error) male and female worms **(A)**, eggs per gram (EPG) of liver **(B),** and eggs per female worm **(C)** recovered from infected wild type (WT) and human microbiota-associated (HMA) mice at 50 days post cercarial exposure. Horizontal lines represent significant differences between mouse lines: *p < 0.05; **p < 0.01.

All HMA mice of both experimental batches were successfully infected with *S. mansoni*, whereas no adult worms or eggs were recovered from two *Sm*-exp WT animals included in the second experiment at *post mortem*; the latter were thus removed from subsequent analyses (**Table 1**). Significantly higher worm burdens (p=0.017, t=2.67, df=16) and EPG of liver (p=0.008, t=3.02, df=16) were observed in HMA compared to WT mice (cf. **Figures 1A, B**; cf. **Supplementary Table 1**). Since differences in EPG of liver could be explained by higher worm burdens observed in HMA mice (**Figure 1C**), changes to parasite fecundity are unlikely. No significant differences were observed in the number of EPG of liver per female between the two groups of mice (p=0.897, U=38.00; **Supplementary Table 1**). Furthermore, the higher infection burdens observed in HMA compared to WT mice were accompanied by significantly higher levels of *Schistosoma*-specific antibodies in sera from HMA animals (**Supplementary Figures 1** and **2**).

### Diversity and Compositional Traits Differentiate the Gut Microbial Profiles of WT and HMA Mice at Baseline

**Figure 2:**
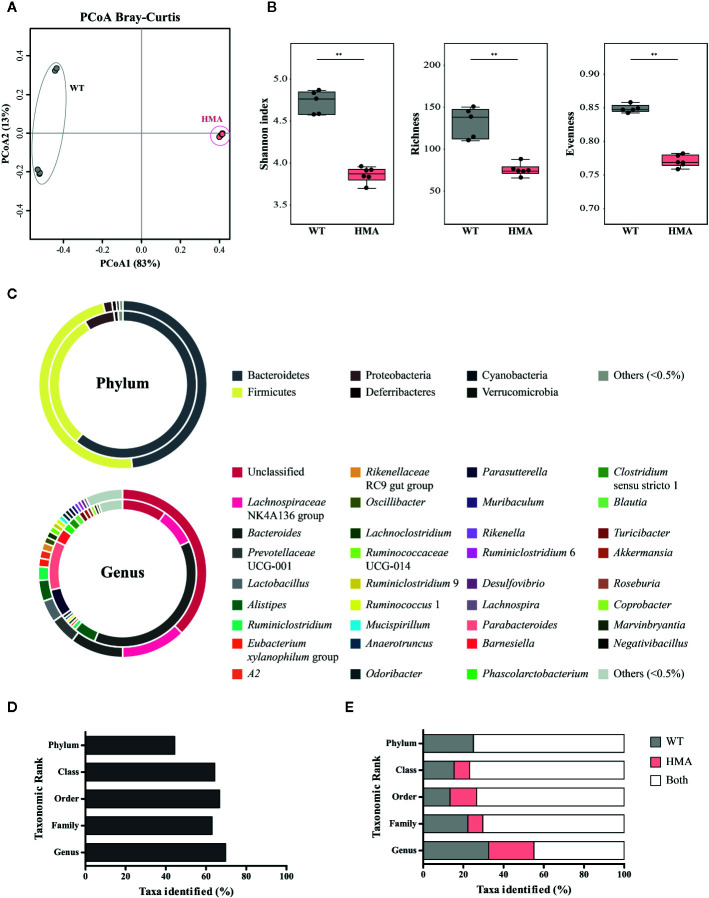
The composition of the baseline gut microbiota differs extensively between naïve wild type (WT) and human microbiota-associated (HMA) mice. **(A)** Principal Coordinate Analysis (PCoA) performed at amplicon sequence variant (ASV) level. **(B)** Differences in microbial alpha diversity; horizontal lines represent significant differences between mouse lines: **p < 0.01. **(C)** Doughnut charts representing the mean relative abundances (TSS-transformed data) of gut microbial phyla and genera identified in feces of naïve WT (outer ring) and HMA (inner ring) mice. Others = sum of all taxa individually representing >0.5% of the overall microbial community. Unclassified = sum of all unclassified genera. **(D)** Percentage of bacterial taxa (from phylum to genus) for which significant differences were detected between the gut of naïve WT and HMA mice, according to both LEfSe and ANOVA (see differentially abundant taxa in **Supplementary Table 2**). **(E)** Percentage of taxa identified (from phylum to genus) solely detected in the gut microbiota of either WT or HMA mice, and in both rodent lines.

**Figure 3:**
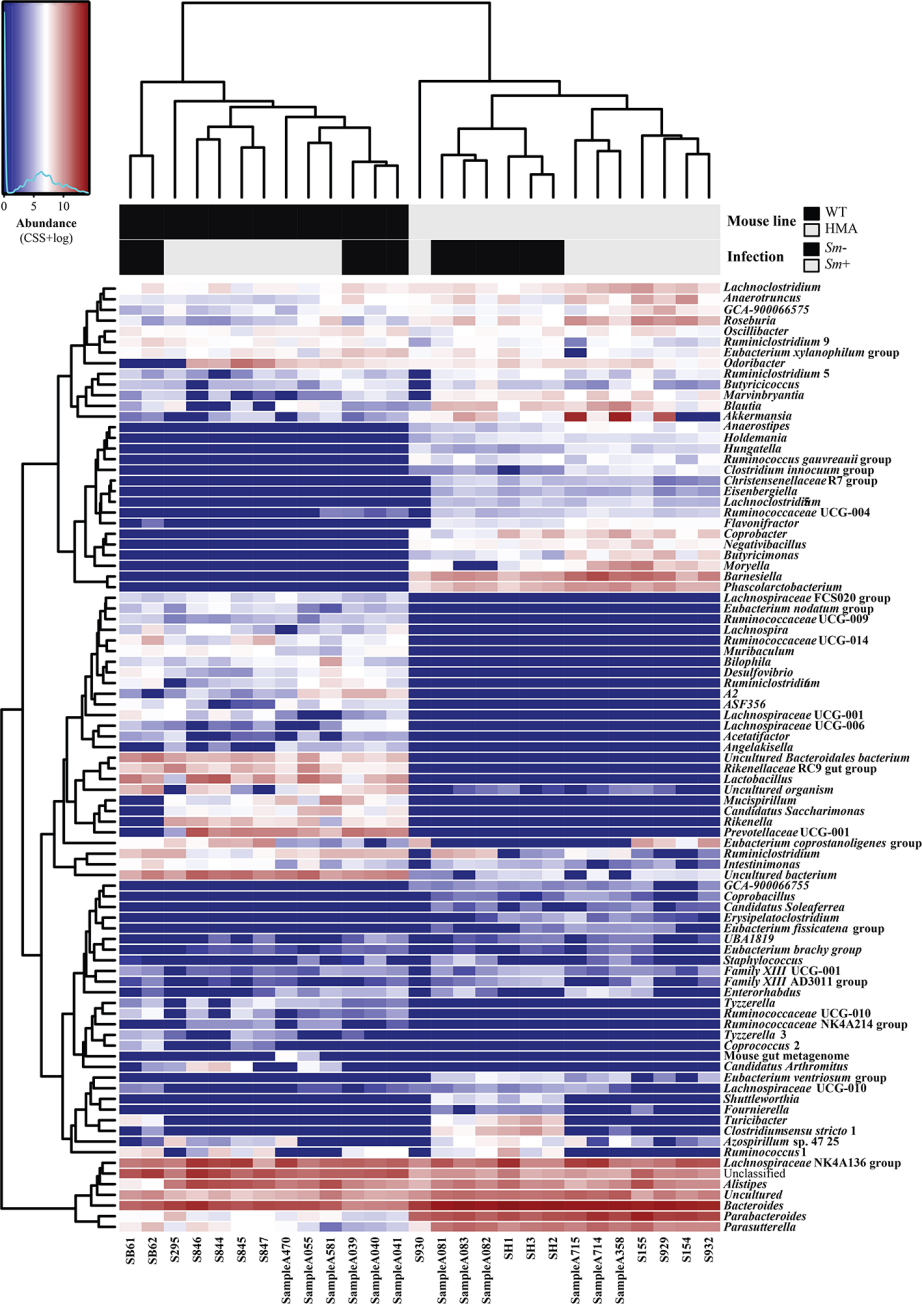
Microbial community composition of all fecal samples from *Schistosoma mansoni*-infected (*Sm*+) and uninfected (*Sm*-) wild type (WT) and human microbiota associated (HMA) mice analyzed in this study, at genus level. In the heatmap, columns represent samples and rows represent genera abundance, both ordered by hierarchical clustering. Explanatory variables (i.e. mouse line and infection status) are presented as a separate heatmap at the top of the figure.

The two rodent lines employed in this study were characterized by marked differences in (i) the origin of the microbiome (wildtype *vs.* heterologous from a human donor) and (ii) exposure to environmental microbes (maintained under specific-pathogen free [SPF] conditions *vs*. in isolation). We anticipated that these profound dissimilarities might significantly impact the composition of the gut microbiota of each mouse line; therefore, we first investigated structural and compositional differences between the gut microbial communities of these rodent lines at baseline. Fecal microbial profiles obtained from samples from *Sm-* WT and HMA were clustered separately by PCoA, which led to the identification of a putative outlier sample in the former group (**Supplementary Figure 3**). Dixon’s Q test for outliers, calculated on the Shannon diversity of all *Sm-* WT mice, supported this hypothesis (p=0.031; Q=0.66), and thus sequence data obtained from this sample were removed prior to further analyses (**Table 1**). PCoA analysis of gut microbial profiles of uninfected WT and HMA mice revealed profound differences between the gut microbiota compositions of these rodent lines (cf. **Figure 2A**). Notably, the gut microbiota composition of HMA mice was highly homogenous, unlike that of WT mice (**Figure 2A**). This observation was confirmed by ANOSIM, which revealed a significantly higher Bray-Curtis dissimilarity among WT compared to HMA mice (p=0.004, R=1; **Supplementary Figure 4**). Significant differences in fecal microbial alpha diversity were also detected between uninfected WT and HMA (**Figure 2B**), with significantly reduced microbial richness (p=0.004, U=0.0; **Figure 2B**) and evenness (p=0.004, F=0.0; **Figure 3B**) observed in the fecal microbiota of HMA compared to WT mice (**Figure 2B**).

In addition to differences in microbial community structure, significant dissimilarities were observed between the taxonomic composition of the gut microbiota of WT and HMA mice prior to parasite infection (**Figures 2C–E** and **Supplementary Table 2**). The phyla Bacteroidetes and Firmicutes were most abundant in the gut of both mouse lines (**Figure 2C**). However, in the gut microbiota of WT animals, similar proportions of these two phyla were observed (measured as mean TSS-transformed relative abundance ± standard deviation), i.e. 48.21 ± 3.61% Bacteroidetes compared with 47.94 ± 3.84% Firmicutes. In contrast, the phylum Bacteroidetes predominated in HMA mice, with 61.52 ± 3.52% compared with 30.73 ± 3.79% Firmicutes. Proteobacteria was the third most represented bacterial phylum in both rodent lines (1.72 ± 0.98% in WT and 6.70 ± 1.72% in HMA mice), followed by Deferribacteres and Cyanobacteria in WT (0.86 ± 1.23% and 0.58 ± 0.64%, respectively), and Verrucomicrobia in HMA (0.95 ± 0.93%; **Figure 2C**). No Deferribacteres or Patescibacteria were detected in the gut microbiota of HMA mice (**Figure 2C**). At genus level, the *Lachnospiraceae* NK4A136 group was most abundant in the gut of WT animals (12.41 ± 2.55%), followed by the genera *Bacteroides* (10.23 ± 3.62%), *Prevotella* UCG-001 (5.11 ± 4.84%) and *Lactobacillus* (4.28 ± 3.50%). In contrast, the genus *Bacteroides* was predominant in the gut of HMA mice (38.31 ± 3.69%), followed by *Parabacteroides* (10.82 ± 0.94%), *Lachnospiraceae* NK4A136 group (8.19 ± 4.89%) and *Parasutterella* (6.12 ± 1.75%) (**Figure 2C**). Furthermore, large differences in the proportions of unclassified microbial genera were observed between mouse lines, together representing 37.56% of the fecal microbial communities in WT animals *vs*. the 9.73% in HMA mice (**Figure 2C**).

Differences in the abundances of individual microbial taxa in the gut of uninfected WT and HMA mice were assessed by LEfSe and ANOVA on CSS+log transformed 16S rRNA sequence data (**Figures 2D, E** and **Supplementary Table 2A, B**). In particular, significant differences (LEfSe: LDA score (log10)>3; ANOVA p<0.05) were detected in ~45% of the phyla, and >60% of the microbial classes, orders, families and genera composing the gut microbiota of the two mouse lines (**Figure 2D**; **Supplementary Table 2**). Among these, the genera *Eubacterium coprostanoligenes* group and *Oscillibacter*, both belonging to the family *Ruminoccocaceae*, were significantly more abundant in the gut of WT mice, while several genera, including *Blautia*, *Roseburia, Clostridium* sensu stricto 1, *Bacteroides* and *Parabacteroides*, among others, showed greater abundances in HMA animals (**Supplementary Table 2**).

Additionally, a large number of bacterial taxa were solely detected in either WT or HMA (**Figure 3**; **Supplementary Table 2**). For instance, at genus level, >50% of the taxa identified in this study were exclusively detected in either WT or HMA mice (**Figure 2E**). Of these, *Prevotellaceae* UCG-001 (5.11% ± 4.84%) and *Lactobacillus* (4.28% ± 3.50%) were exclusively detected in WT, while *Barnesiella* (3.00 ± 1.10%) in HMA mice (**Figures 2C** and **3**). However, the majority of these taxa were low-abundant bacteria, each representing <1% of the overall gut microbial population. In particular, numerous ‘line-exclusive’ genera belonged to the family *Lachnospiraceae* (**Supplementary Table 2**), the second most abundant family in the gut of both mouse lines. Furthermore, the orders Deferribacterales and Saccharimonadales, along with the families *Lactobacillaceae* and *Muribaculaceae* were detected exclusively in the gut of WT animals, whereas the orders Slenomonadales and DTU014 were solely found in the gut of the HMA hosts (**Supplementary Table 2**).

### *Schistosoma mansoni* Infection Is Associated With Differences in Gut Microbiota Alterations Between HMA and WT Mice

**Figure 4:**
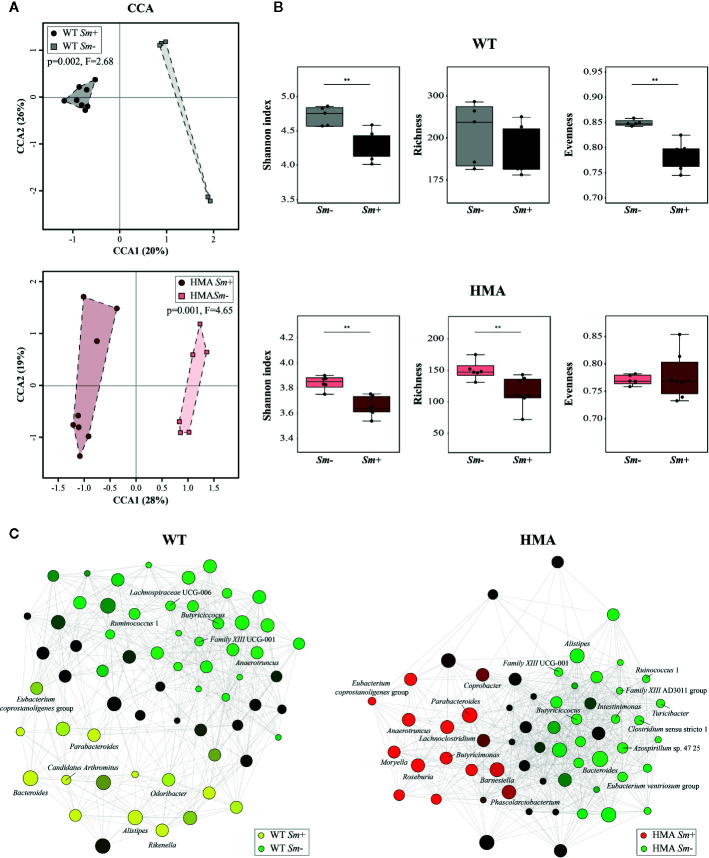
Infection by *Schistosoma mansoni* is associated with significant alterations of the fecal microbiota composition of wild type (WT) and human microbiota-associated (HMA) mice. **(A)** Canonical Correlation Analysis (CCA) of microbial profiles of WT and HMA mice, performed at amplicon sequence variant (ASV) level and clustered according to infection status [i.e. infected (*Sm+*) *vs*. uninfected (*Sm*-)]. **(B)** Differences in microbial alpha diversity between the fecal microbial profiles of infected and uninfected animals in each rodent line; horizontal lines represent significant differences between rodent lines: **p < 0.01. **(C)** Clustering of microbial genera co-occurring in the fecal microbiota of infected and uninfected WT and HMA mice (the full list of differentially abundant taxa is available from **Supplementary Table 3**).

The extensive differences observed between the gut microbiota composition of HMA and WT mice at baseline prevented us from conducting direct comparisons between infected and uninfected animals of these rodent lines; therefore, we next investigated the impact of *S. mansoni* infection on the gut microbiota profile of each host. Colonization by *S. mansoni* was associated with overall changes in the gut microbiota composition of both WT and HMA mice, as determined by CCA (WT: p=0.002, F=2.68; HMA: p=0.001, F=4.65; **Figure 4A**). In both mouse lines, these changes included a significant decrease in microbial alpha diversity post-infection, as well as variations in the abundances of selected populations of gut bacteria (**Figure 4**, **Supplementary Figure 5** and **Supplementary Table 3**). Notably, the reduction in microbial alpha diversity observed in *Sm*+ WT mice (Shannon index [ASV], p=0.006, U=2.0) was associated with a significant decrease in evenness (p=0.002, U=0.0), whereas that observed in *Sm*+ HMA mice (Shannon index [ASV], p=0.008, U=4.0) was linked to a significant decrease in ASV richness (p=0.005, U=3.0) (**Figure 4B**).

Variations in the abundances of individual microbial taxa in the gut of WT and HMA mice following infection by *S. mansoni* were assessed by LEfSe and ANOVA on CSS+log transformed 16S rRNA sequence data (**Supplementary Table 3A–C**). Furthermore, network analyses led to the identification of populations of gut bacteria associated with infection by *S. mansoni* in each mouse line (**Figure 4C**), while information on the abundances of individual microbial genera across all samples is shown in **Figure 3**. At day 50 p.i. significant alterations in the abundances of several families and genera belonging mainly to the orders Clostridiales and Bacteroidales were observed in *Sm+* WT and HMA mice, compared to *Sm-* (**Supplementary Table 3**). Within the Clostridiales, members of the family *Lachnospiraceae*, such as *Lachnospiraceae* UCG-006 and UCG-10, were significantly reduced in the gut microbiota of WT mice following infection, while *Lachnospiraceae* FCS020 group was more abundant in the gut of these animals (**Figure 4C** and **Supplementary Table 3**). In the HMA line, selected *Lachnospiraceae*, i.e. *Shuttleworthia* and *Eubacterium ventriosum* group, were significantly reduced in the gut microbiota of infected animals, whereas other genera, including *Moryella*, *Roseburia* or *Lachnoclostridium*, were significantly expanded (**Figure 4C** and **Supplementary Table 3**). Similarly, several members of the family *Ruminococcaceae* were either reduced or expanded in the gut microbiota of each mouse line post-infection (**Supplementary Table 3**). For instance, the genus *Anaerotruncus* was negatively associated with *Sm*+ WT mice based on both ANOVA and network analyses; in contrast, a positive correlation between the abundance of this bacterial genus and helminth infection was observed in HMA rodents by both LEfSe and network analyses (**Figure 4C** and **Supplementary Table 3**). The family *Clostridiaceae* 1 was significantly associated with infected and uninfected WT and HMA mice, respectively. In WT, this finding was linked to a significant expansion of the genus *Candidatus Arthromitus* following *S. mansoni* infection, while a significant contraction in populations of *Clostridium* sensu stricto 1 was detected in *Sm*- HMA mice (**Figure 4C** and **Supplementary Table 3**).

Among members of Bacteroidales whose abundances were altered following *S. mansoni* infection, the genus *Parabacteroides* was associated with helminth colonization in both mouse lines by network analysis (**Figure 4C**); however, LEfSe and ANOVA only detected significant infection-associated increases in populations of *Parabacteroides* in HMA animals (**Supplementary Table 3**). Furthermore, while significant expansions of populations of *Bacteroides* and *Alistipes* were observed in the gut of infected WT mice (**Figure 4C** and **Supplementary Table 3**), network analysis revealed a negative association between these genera and infection by *S. mansoni* in HMA animals (**Figure 4C**). Nevertheless, significant differences in intestinal populations of *Bacteroides* or *Alistipes* were not detected between *Sm+* and *Sm*- HMA mice by either LEfSe or ANOVA (**Supplementary Table 3**). In addition, within the Bacteroidales, LEfSe and network analyses detected positive associations between the genera *Odoribacter* and *Rikenella* (the latter also supported by ANOVA) and *S. mansoni* infection in WT mice (**Figure 4C** and **Supplementary Table 3**). In HMA mice, however, infection was associated with significant expansions of bacteria within the family *Barnesiellaceae*, and the related genera *Barnesiella* and *Coprobacter* (detected by LEfSe and network analysis), as well as the genus *Butyricimonas* (detected by LEfSe, ANOVA and network analysis) (**Figure 4C** and **Supplementary Table 3**); notably, these taxa were solely detected in the gut of HMA mice (**Supplementary Table 2**).

Besides changes in the abundances of members of the Clostridiales and Bacteroidales, *S. mansoni* infection was associated with significant alterations in populations of Alphaproteobacteria, and of the genus *Azospirillum* sp. 47 25 in particular, which was positively and negatively linked to parasite colonization in WT and HMA mice, respectively (**Figure 4C** and **Supplementary Table 3**). Furthermore, in HMA animals, helminth infection was linked to significant reductions in populations of the class Bacilli (and order Bacillales), and expansions of the class Negativicutes (order Selenomonadales, family *Acidaminococcaceae* and genus *Phascolarctobacterium*); the latter bacterial class (and related taxa) was solely detected in the gut microbiota of HMA mice (**Supplementary Table 3**).

For each rodent line, correlations between the abundances of individual gut bacteria and infection burdens (i.e. worm counts and EPG of liver) were investigated by multiple regression. While the genera *Lachnospiraceae* UCG-006 and *Family XIII* UCG-001 were negatively correlated with worm counts and EPG of liver in WT mice, the abundances of *Bacteroides* and *Rikenella* were positively correlated with infection burdens (worm counts and/or EPG of liver) in *Sm*+ WT hosts (**Supplementary Table 4**). In contrast, in HMA mice, parasite burdens were negatively correlated with fecal populations of *Turicibacter*, *Shuttleworthia*, *Ruminococcus* 1 and *Clostridium* sensu stricto 1, and positively correlated with those of *Moryella* and *Lachnoclostridium*, among other genera. The complete list of bacterial genera whose abundances correlated with infection burdens in either line is available from **Supplementary Table 4**. These findings were consistent with data obtained by LEfSe (**Supplementary Table 3A**), ANOVA (**Supplementary Table 3B, C**) and/or taxa correlation network analyses (**Figure 4C**).

### Fecal Bacteria Correlated With Infection Burdens in Both WT and HMA Mice

While the study of the alterations of the gut microbial composition associated with *S. mansoni* infection in HMA and WT mice revealed substantial differences between rodent lines, some common traits were identified. In order to identify fecal microbial taxa consistently associated with infection in both mouse lines, the LEfSe workflow was applied to all metagenomics datasets, while controlling for both infection status and rodent line. In accordance with the findings obtained from individual analyses of each rodent line, the genus *Eubacterium coprostanoligenes* group was positively associated to *S. mansoni* infection when the two variables (i.e. ‘infection status’ and ‘mouse line’) were considered, whereas the genera *Ruminococcus* 1, *Butyricicoccus* and *Family XIII* UCG-001, among others, were associated to *Sm*- samples (**Supplementary Table 5**). Correlations between the fecal abundances of key bacterial genera identified by LEfSe with worm counts and EPG of liver were investigated by multiple regression analysis. Significant negative associations were detected between infection burden and fecal abundances of *Ruminococcus* 1, *Butyricicoccus*, *Family XIII* UCG-001 and *Family XIII* AD3011 in both rodent lines, while a positive correlation was observed between the abundance of *Eubacterium coprostanoligenes* group and total worm counts (**Supplementary Table 5B**). Linear regression data of worm burdens/EPG of liver *vs.* taxa abundance in the two rodent lines showed similar trends to those observed in each WT and HMA (**Supplementary Figure 6**).

## Discussion

Animal models of infection are used extensively to address causal relationships between helminth colonization, alterations in gut microbial composition, and changes in the immune and/or metabolic functions of the host (12). Nevertheless, helminth-gut microbiota interactions are highly heterogeneous depending on the host-parasite pair under consideration (4). Thus, comparing data between different host-parasite systems, even when these involve the same helminth species, may lead to misinterpretations. On the other hand, studies on naturally infected humans may provide data on associations between gut microbial composition and infection with one or more parasites (33); nevertheless, such studies often lack proof of causality between gut microbial signatures and susceptibility to, or pathophysiology of, infection (34, 35). Therefore, complementary studies involving naturally-infected human populations in endemic areas and experimental animal models are pivotal to advance our knowledge of the contribution of the gut microbiota in human parasitic diseases. Towards this aim, novel animal models of infection involving mice colonized by gut microbes of human origin (i.e. HMA mice) may offer unique research opportunities. Thus far, these experimental systems have been applied to investigations of the contribution of human dysbiotic microbiomes to immune- and metabolic-mediated diseases such as diabetes, obesity, inflammatory bowel disease and cancer (34). In this study, HMA mice were used to explore the links between *S. mansoni* infections and host gut microbiota composition. To the best of our knowledge, this is the first study investigating the relationships between helminth infections and the gut microbiota of the definitive host *via* the use of HMA mice.

Furthermore, prior to our study, no data was available on the associations between the baseline composition of the host gut microbiota and host susceptibility to *Schistosoma* colonization and/or pathogenicity. Signatures of gut microbial dysbiosis (i.e. expanded Proteobacteria) have been associated with *S. haematobium* infection in adolescents from an endemic region (17), as well as to overt clinical signs of schistosomiasis mansoni (i.e. blood in stool and/or splenomegaly) and adverse effects to praziquantel treatment in children (16). However, it remains unclear whether dysbiosis represents an outcome of infection or a risk factor leading to the development of severe disease in young susceptible individuals (17).

In this study, associations between the outcome of *S. mansoni* infections and the gut microbiota composition at the time of infection were detected in WT and HMA mice. The baseline gut microbiota profile of HMA mice is influenced by multiple evolutionary and ecological factors that impact on the colonization, establishment and growth of selected groups of bacteria (among other microbes) at the expense of others, generally resulting in intestinal dysbiosis (34). Interestingly, in our study, HMA mice were colonized by higher worm burdens – resulting in higher egg loads in the liver – and displayed an enhanced production of anti-*S. mansoni* SEA antibodies than WT animals, suggesting the occurrence of an association between baseline gut microbiota composition and susceptibility to parasite infection. Of note, gut microbial dysbiosis in naïve HMA mice was characterized by enlarged proportions of Proteobacteria and low microbial richness and overall alpha diversity (compared to WT animals), which is partially consistent with data from studies performed in humans from endemic areas, where no alterations in alpha diversity had been reported (16, 17).

While the mechanisms underpinning the higher worm and egg burdens observed in HMA hosts are yet to be discovered, one hypothesis might be linked to the modified immune system that characterizes these mice. Indeed, the gut microbiome of vertebrate animals is essential for the maturation and proper functioning of their immune system (36, 37). Germ-free mice are known for their deficient innate and adaptive immunity (38) that, in turn, had been linked to enhanced susceptibility to *S. mansoni* colonization (39). Similarly, HMA mice have been reported to display altered immune responses and susceptibility to intestinal bacterial infections (38, 40, 41). In humans, gut microbial dysbiosis has been associated with a number of pathological conditions (42); importantly, in resource-limited settings, such as those where schistosomiasis is endemic (13), young children are often affected by environmental enteric dysfunction, a multifactorial disorder associated with early childhood malnutrition, growth retardation and impaired immune function (43, 44). Intestinal dysbiosis is key to the pathogenesis of environmental enteric dysfunction (43, 44); therefore, causal relationships may occur between gut microbial imbalances in children from these areas and susceptibility to schistosomiasis that deserve to be thoroughly investigated.

The large number of bacterial taxa that were solely detected in either HMA or WT mice is noteworthy and, together with the substantial differences in the relative proportions of several other bacteria detected between the two rodent lines, might be linked to the different burdens of *S. mansoni* observed in these experiments. Indeed, altered populations of immune-modulatory bacteria (i.e. lactobacilli) in the mouse gut have been linked to differences in immune responses and susceptibility to infection by the gastrointestinal helminth *Heligmosomoides polygyrus* (6). In our study, *Lactobacillaceae* (including the genus *Lactobacillus*) were only detected in the gut of WT mice, although populations of these bacteria were not altered by infection. Previously, transient expansions of *Lactobacillus* were reported in the gut of Swiss-Webster mice exposed to large numbers of *S. mansoni* cercariae (19). The role of lactobacilli in regulating *S. mansoni* infection remains, however, unknown, and further studies are necessary in order to ascertain whether the absence of these bacteria in the gut of HMA mice might be linked to the higher infection burdens observed in these animals.

The genus *Barnesiella* was solely identified in the feces of HMA mice, and was significantly expanded in the gut of infected animals. While members of this genus have been regarded as immunomodulatory, current evidence is inconsistent. Expanded populations of *Barnesiella* spp. have been linked to Th1 environments in diverse murine models (45–47). In contrast, in humans, negative correlations between fecal abundances of *Barnesiella* and the ability of peripheral blood mononuclear cells to produce IFN-γ following stimulation with either lipopolysaccharide or *Bacteroides fragilis* have been described (48). IFN-γ has been reported to mediate protection against *S. mansoni* infection in vaccine studies (49–51). Moreover, Th1 responses are dominant during pre-patent schistosomiasis (52). Therefore, given the known links between *Barnesiella* and Th1-mediated immunity in mice and humans, the potential role(s) of this genus of bacteria in modulating the response against *S. mansoni* in HMA mice merit(s) further investigations.

Structural and compositional differences between the gut microbiota of WT and HMA mice at baseline might explain the large dissimilarities observed in infection-associated changes in microbial profiles. However, some common traits were noted. For instance, the genus *Ruminococcus* 1 (the abundance of which was negatively correlated with infection burdens in both WT and HMA mice) includes mucin-degrading bacteria (53, 54) whose growth might be affected by variations in mucosal carbohydrate availability (55). Alterations in intestinal mucus production and glycosylation are known to occur during gastrointestinal helminth infections (56–58) and, given the essential role of the IL-4/IL-13 pathway in goblet cell hyperplasia, mucus secretion and glycosylation (59), are likely to arise during intestinal schistosomiasis. Therefore, it is plausible that reduced populations of *Ruminococcus* 1 in the gut of *Sm*+ mice might be linked to changes in mucin glycan composition; this, in turn, might be associated to egg-induced immunity and disruption of the intestinal barrier. On the other hand, selected *Ruminococcus* spp. play roles in the breakdown of dietary polysaccharides and production of immunomodulatory short-chain fatty acids (SCFAs) (60, 61). Other populations of SCFAs-producing bacteria, such as *Butyricicoccus* (62), and the genera *Shuttleworthia* (63) and *Intestinimonas* (64, 65), were also negatively correlated with *S. mansoni* infection burdens (the latter two in HMA mice only); thus, reductions in the abundances of these populations of gut bacteria might lead to an impaired regulation of intestinal immunity and barrier function in infected animals (66).

While these findings suggest the occurrence of associations between changes in gut microbiota composition and alterations of host immune and intestinal barrier function over the course of schistosomiasis, the profound differences between the gut microbial profiles of HMA and WT mice call for caution when selecting bacterial candidates for follow-up experiments aimed at ascertaining the role of the host gut microbiota in modulating immunity to infection. For instance, despite the abovementioned reduction in several SCFA-producing bacteria in the intestines of both *Sm+* WT and HMA mice, butyrate-producing *Roseburia* (67) were significantly more abundant in the gut of HMA hosts at baseline, and were further expanded post schistosome infection. Hence, similar changes in gut microbial composition of both lines might not result in comparable immune responses in each host. Nevertheless, laboratory rodent lines characterized by different susceptibility to infection, and with predefined profiles of immunomodulatory gut bacteria might be useful to investigate the contribution of the host gut flora to resistance to *Schistosoma* infection *via*, for instance, fecal transplants or gut recolonization with selected bacteria (68). In particular, HMA mice might represent invaluable models for mechanistic studies aimed to reveal causal relationships between alpha diversity and/or selected populations of immunomodulatory bacteria (and other microscopic inhabitants of the gut including prokaryotes, eukaryotes and viruses) and immunity to infection.

Furthermore, given that HMA mice are derived from germ-free animals and maintained under strict conditions of isolation, it is tempting to speculate that, besides the gut microbiota, microbial communities colonizing other organs such as the skin or the respiratory tract might also be dysbiotic. The skin microflora plays important roles in shaping cutaneous immunity and preventing pathogen colonization (69), while interactions between the gut and lung microbiome (i.e. the so-called gut-lung axis) have been linked to alterations of the lung immune function (70). The contributing roles of skin and lung microbiomes in regulating immunity to and survival of schistosomulae are yet to be investigated; this, together with the current lack of data on the composition of the dermal and respiratory microbiota of HMA mice, prevents us from further speculating about the potential role(s) of skin and/or lung dysbiosis in enhanced susceptibility to *S. mansoni* infection. Nevertheless, our results suggest that HMA mouse models could be useful to test the contribution of such microbial communities to the defense against the juvenile stages of *Schistosoma* spp.

## Conclusions and Future Perspectives

Overall, our results suggest that the baseline composition of the host gut microbiome might impact host susceptibility to *S. mansoni* colonization, as well as infection-associated changes in gut microbial profiles. However, further studies are necessary to ascertain the impact that gut microbial dysbiosis (i.e. reduced alpha diversity) and/or selected human microbes exerts on *S. mansoni* infection rates. In particular, such studies should include (i) experimental infections of additional HMA mouse lines, (ii) groups of control rodents derived from germ-free mice transplanted with the fecal microbiota of WT mice, and (iii) antibiotic-treated WT mice. Moreover, predictive models linking infection burdens to the abundances of key gut microbes and/or alpha diversity prior to infection could be useful to identify gut microbial features that might facilitate parasite colonization. This knowledge will underpin future mechanistic studies to untangle the complex interactions between the gut microbiome, the host immune response and the outcome of schistosome infection.

## Data Availability Statement

The datasets presented in this study can be found in online repositories. The names of the repository/repositories and accession number(s) can be found in the article/**Supplementary Material**.

## Ethics Statement

The animal study was reviewed and approved by Animal Welfare and Ethical Review Body (AWERB).

## Author Contributions

ACor, GR and CC designed the research. ACor, SC, ACos, CM, KH, CB, CT, and GR carried out the research. ACor, ACos, AA and JR performed data processing and analyses. ACor, GR, and CC wrote the main manuscript text with input from AM, TL, and MB. The figures were prepared by ACor and ACos. All authors reviewed the manuscript. All authors contributed to the article and approved the submitted version.

## Funding

ACor is supported by Red de Investigación Colaborativa en Enfermedades Tropicales—RICET (RD16/0027/0023—Ministerio de Ciencia, Innovación y Universidades, Madrid, Spain). Wellcome provided core-funding support to the Wellcome Sanger Institute, award number 206194. JR is the grateful recipient of a PhD scholarship by the UK Biotechnology and Biological Sciences Research Council (BBSRC). Research in the CC laboratory is funded by grants by the Isaac Newton Trust and the University of Cambridge.

## Conflict of Interest

The authors declare that the research was conducted in the absence of any commercial or financial relationships that could be construed as a potential conflict of interest.

## Supplementary Material

Supplementary Figure 1 IgG responses to *Schistosoma mansoni* soluble egg antigen (SEA) in the sera of wild type (WT) and human microbiota-associated (HMA) mice. Lines connect samples collected from individual mice from two independent batches of infection (i.e., B1 = dashed, and B2 = solid). OD = Optical density. Asterisks indicate statistically significant differences between the sera of infected (*Sm*+) and uninfected (*Sm*-) mice (*p<0.05).

Supplementary Figure 2 IgM responses to *Schistosoma mansoni* soluble egg antigen (SEA) and total IgE in the sera of wild type (WT) and human microbiota-associated (HMA) mice. Lines connect samples collected from individual mice from two independent batches of infection (i.e., B1 = dashed, and B2 = solid). OD = Optical density. Asterisks indicate statistically significant differences between the sera of infected (*Sm*+) and uninfected (*Sm*-) mice (*p<0.05).

Supplementary Figure 3 Principal Coordinate Analysis (PCoA) performed at amplicon sequence variant (ASV) level (A) and estimated microbial alpha diversity (B) of microbial profiles of uninfected wild type mice (WT); red circles indicate the outlier sample identified in this experimental group, which was removed prior to further downstream processing. (C) PCoA (ASV) depicting the microbial profiles of uninfected human microbiota-associated mice (HMA).

Supplementary Figure 4 Microbial beta diversity of uninfected wild type (WT) and human microbiota-associated (HMA) mice. Differences between mouse lines were assessed by ANOSIM on Bray-Curtis dissimilarities (**p<0.01). Between = beta diversity between groups.

Supplementary Figure 5 Gut microbial profiles of wild type (WT) and human microbiota-associated (HMA) mice following infection by *Schistosoma mansoni.* Doughnut charts representing the mean relative abundances (TSS-transformed data) of gut microbial genera detected in the feces of uninfected (outer ring) and *S. mansoni*-infected (inner ring) WT and HMA mice. Others = sum of all genera individually representing less than 0.5% of the overall microbial community. Unclassified = sum of all unclassified genera.

Supplementary Figure 6 Correlations between *Schistosoma mansoni* worm burdens and eggs per gram (EPG) of liver, and abundances of selected bacterial genera in feces of wild type (WT) and human microbiota associated (HMA) mice.

## Abbreviations

ASV, Amplicon Sequence Variant; EPG, Eggs per gram; HMA, Human microbiota-associated; SCFA, Short Chain Fatty Acid; SEA, Soluble egg antigen; *Sm*-exp, Exposed to *Schistosoma mansoni* cercariae; *Sm-*, Uninfected; *Sm+*, Infected with *Schistosoma mansoni;* WT, Wildtype.
