# Supplementary figures and images for: Baseline Gut Microbiota Composition Is Associated With Schistosoma mansoni Infection Burden in Rodent Models
Source: Front Immunol. 2020 Nov 18;11:593838. doi: 10.3389/fimmu.2020.593838 (PMC7718013; doi:10.3389/fimmu.2020.593838)

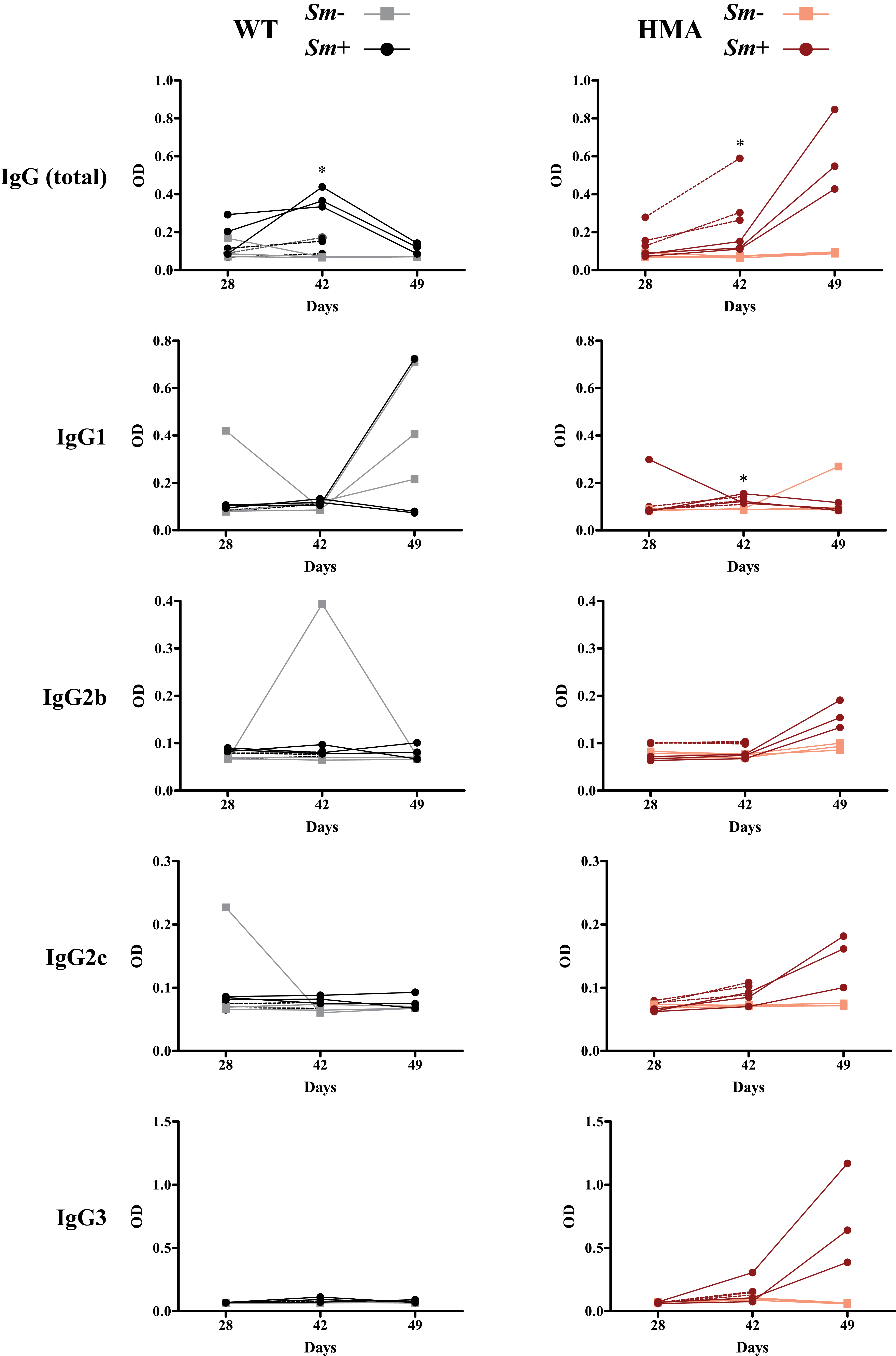

Supplement: Supplementary Figure 1 — IgG responses to Schistosoma mansoni soluble egg antigen (SEA) in the sera of wild type (WT) and human microbiota-associated (HMA) mice. Lines connect samples collected from individual mice from two independent batches of infection (i.e., B1 = dashed, and B2 = solid). OD = Optical density. Asterisks indicate statistically significant differences between the sera of infected (Sm+) and uninfected (Sm-) mice (*p<0.05). [file Image_1.png]

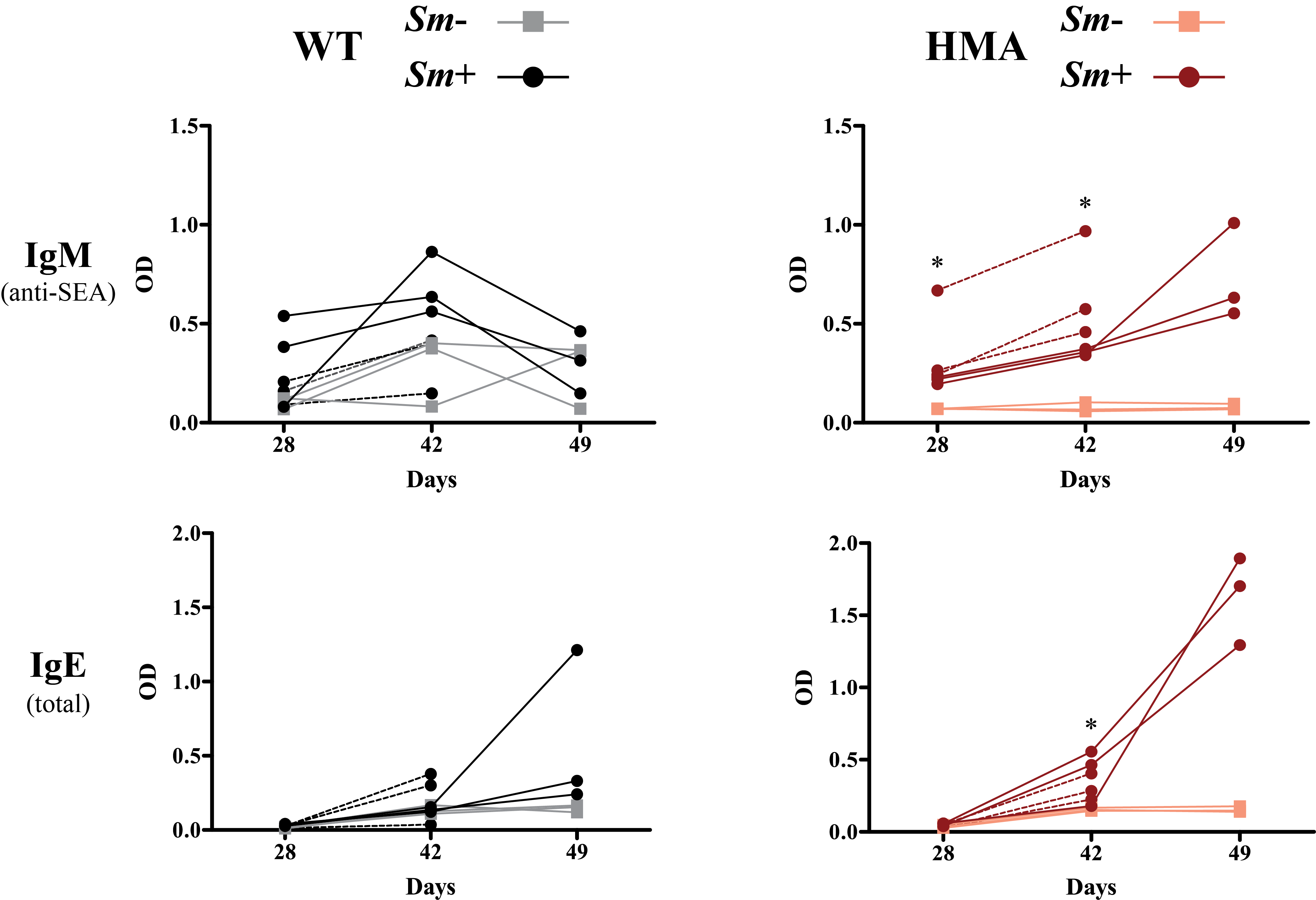

Supplement: Supplementary Figure 2 — IgM responses to Schistosoma mansoni soluble egg antigen (SEA) and total IgE in the sera of wild type (WT) and human microbiota-associated (HMA) mice. Lines connect samples collected from individual mice from two independent batches of infection (i.e., B1 = dashed, and B2 = solid). OD = Optical density. Asterisks indicate statistically significant differences between the sera of infected (Sm+) and uninfected (Sm-) mice (*p<0.05). [file Image_2.png]

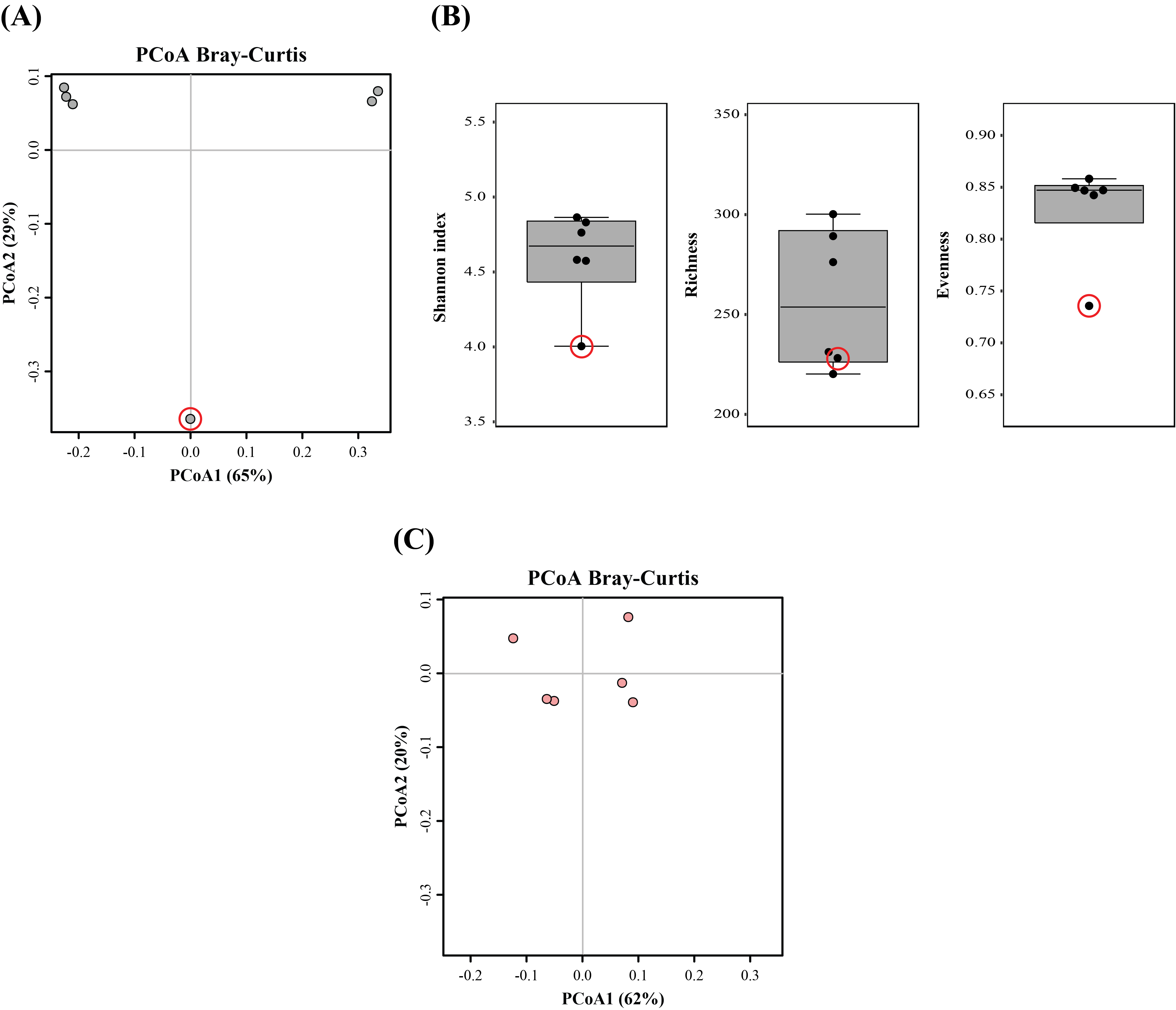

Supplement: Supplementary Figure 3 — Principal Coordinate Analysis (PCoA) performed at amplicon sequence variant (ASV) level (A) and estimated microbial alpha diversity (B) of microbial profiles of uninfected wild type mice (WT); red circles indicate the outlier sample identified in this experimental group, which was removed prior to further downstream processing. (C) PCoA (ASV) depicting the microbial profiles of uninfected human microbiota-associated mice (HMA). [file Image_3.png]

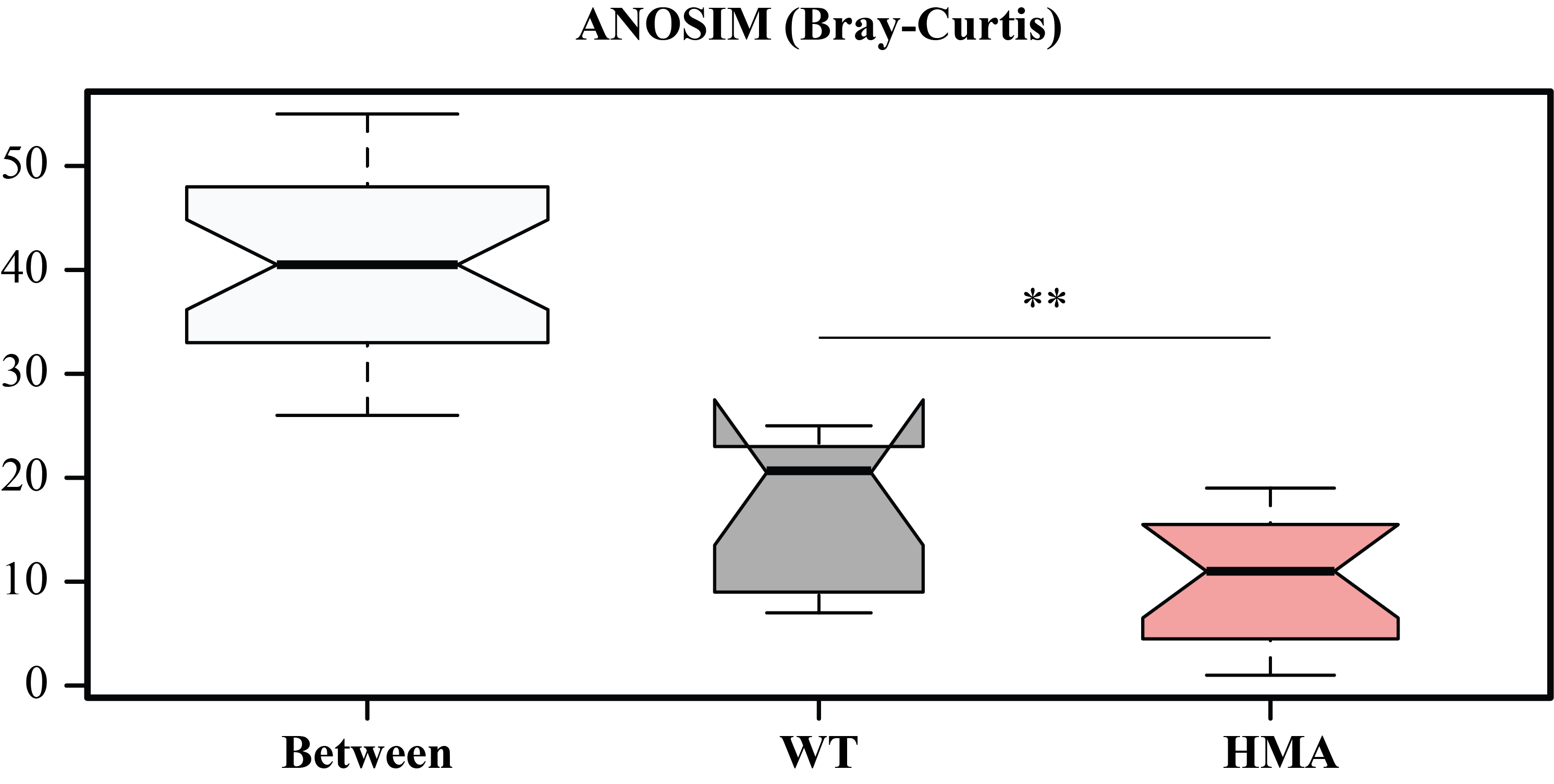

Supplement: Supplementary Figure 4 — Microbial beta diversity of uninfected wild type (WT) and human microbiota-associated (HMA) mice. Differences between mouse lines were assessed by ANOSIM on Bray-Curtis dissimilarities (**p<0.01). Between = beta diversity between groups. [file Image_4.png]

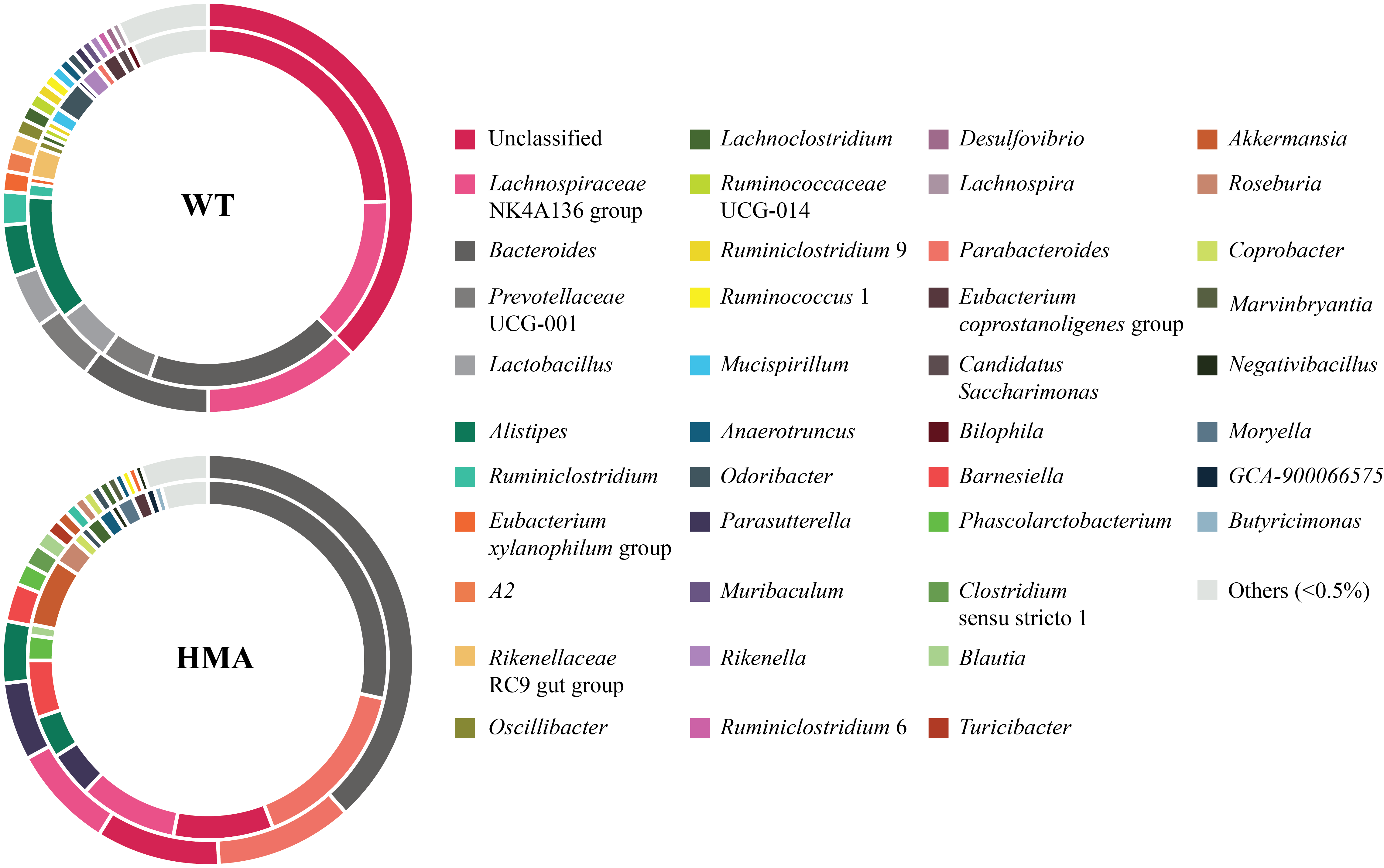

Supplement: Supplementary Figure 5 — Gut microbial profiles of wild type (WT) and human microbiota-associated (HMA) mice following infection by Schistosoma mansoni. Doughnut charts representing the mean relative abundances (TSS-transformed data) of gut microbial genera detected in the feces of uninfected (outer ring) and S. mansoni-infected (inner ring) WT and HMA mice. Others = sum of all genera individually representing less than 0.5% of the overall microbial community. Unclassified = sum of all unclassified genera. [file Image_5.png]

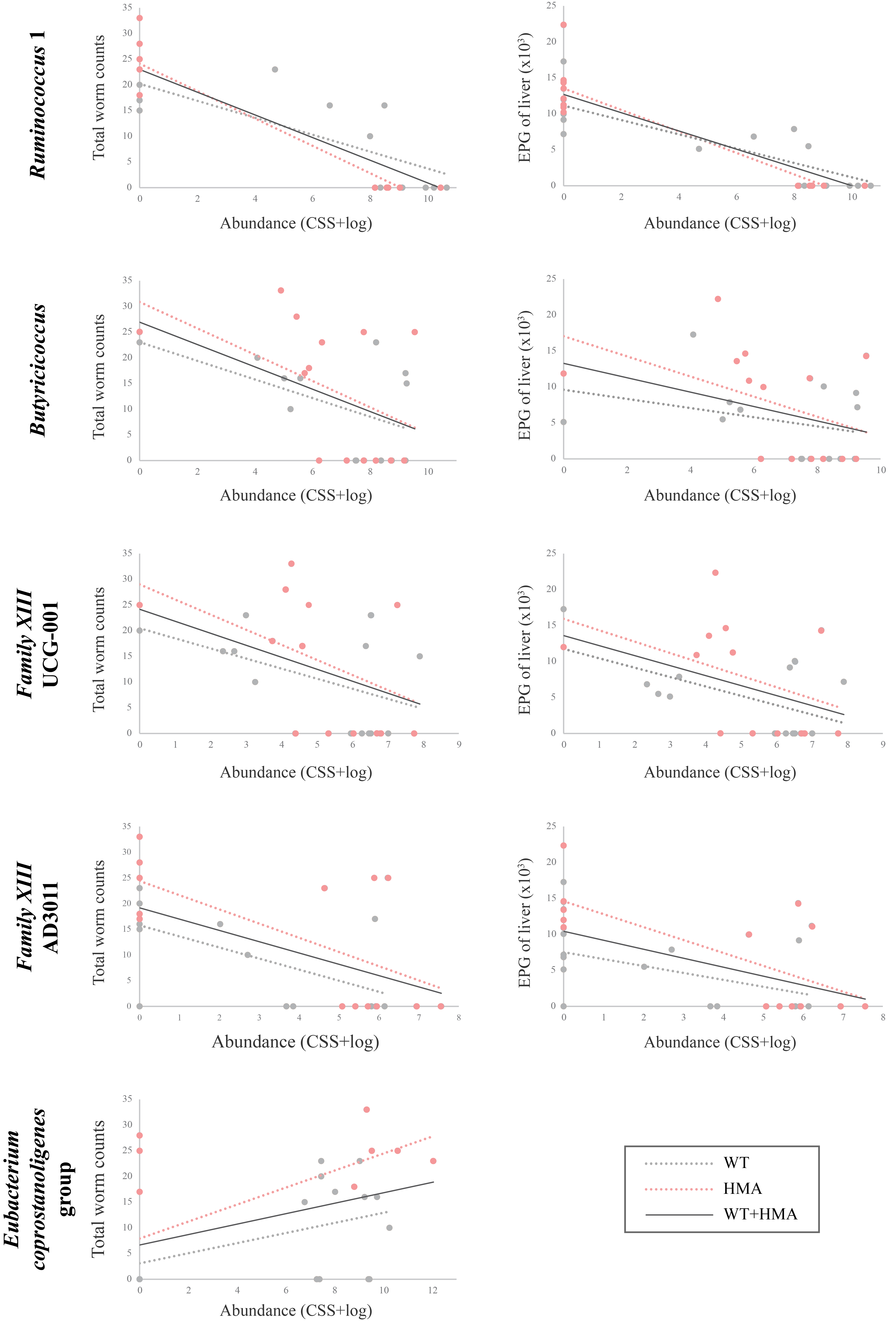

Supplement: Supplementary Figure 6 — Correlations between Schistosoma mansoni worm burdens and eggs per gram (EPG) of liver, and abundances of selected bacterial genera in feces of wild type (WT) and human microbiota associated (HMA) mice. [file Image_6.png]
